# A Sharp Criticism of “Homœopaths” and the Investigator

**Published:** 1884-04

**Authors:** 


### A SHARP CRITICISM OF "HOMŒOPATHS" AND THE INVESTIGATOR.

### THE VIEW OF AN ALLOPATH EXPERIMENTING WITH HOMŒOPATHY.

A New England allopath who is trying Homœopathy writes as follows to a friend in Philadelphia. His criticism is severe, but who can deny its justice?

"In October, I lost a patient with pneumonia. I treated him homœopathically at first. He had complete hepatization of the right lung. On the fourth day I sent for a homœopathic doctor as counsel. What did *he* advise? Drop doses of Tinct. Verat. Viride once in four hours!—when, according to *Lilienthal's Therapeutics*, there was not one symptom indicating Verat. Viride! * * *

"I tell you I have lots of things to be disgusted with. The homœopathic physicians about here are *quacks*. They do not believe in Homœopathy. Take this example: I was called as counsel by Dr. H., of B., in a case of intestinal obstruction. His treatment had been Pil. Cath. Comp., Rhubarb, and Podophyllin in substantial doses. I suggested Bry. or Coloc. These relieved the pain; but on the next day I made a rectal examination and discovered a foreign growth, probably carcinomatous, high up in rectum. The patient died.

"Another thing I am disgusted with is the *Medical Investigator*. What trash! During the year I have read it faithfully, and have seen not more than a dozen articles of value to a student of Homœopathy. At least half of its contributors lack a decent common-school education, as evidenced by their spelling and grammar.

"Take its 'cases for consultation.' Out of a half a dozen replies hardly two will suggest the same drug. Does that look like sharp-shooting? In a medical journal I want the essence of the progressive thought of the leading medical men; in the *Investigator*, I find only the crudest essays of men who are plainly uneducated and incapable of close reasoning. Of course, there are exceptions; I recall a paper on 'Brachial Neuralgia,' and another on 'Sanguinaria Headache,' which were really gems of the first water. Both were, however, extracted from other journals! * * *

"I certainly am not ready to join a homœopathic society comprised of such men as I meet here." B.

